# Classification of gastrointestinal symptom patterns in young adults

**DOI:** 10.1186/s12876-020-01478-7

**Published:** 2020-10-06

**Authors:** Helize Vivier, Emily J. Ross, Jeffrey E. Cassisi

**Affiliations:** Department of Psychology, University of Central Florida, 4111 Pictor Lane, Building 99, Suite 320, Orlando, FL 32816 USA

**Keywords:** PROMIS-GI, DGBI, FGID, Rome IV criteria, Emerging adults, Latent class analysis

## Abstract

**Background:**

The purpose of this study was to identify common gastrointestinal (GI) symptom groups using the Patient-Reported Outcomes Measurement Information System - GI symptom scales (PROMIS-GI) within a large sample of young adults. An attempt was made to relate the emergent groups to the Rome IV disorders of gut-brain interaction symptom domains. The PROMIS-GI is a freely available, adaptable, normatively referenced symptom measurement system that is applicable to many health assessment situations.

**Methods:**

Participants were 956 introductory psychology students between the ages of 18 and 25 who completed the PROMIS-GI as part of ongoing research monitoring physical and psychological health of students at a major southeastern university. GI symptom groups were determined using a latent class analysis (LCA) approach. These GI symptom groups were then compared on key psychosocial factors including self-reported mood, anxiety, and health related quality of life (HRQoL) using MANOVA.

**Results:**

Three groups were identified based on GI symptom elevations: Normal (*n* = 649), Mild (*n* = 257), and Moderate (*n* = 50). Self-reported anxiety, depression, and bodily pain levels were significantly higher in the Mild and Moderate GI symptom groups, and they indicated significantly lower work functioning, and general health ratings compared to participants in the normal group.

**Conclusions:**

Approximately a third of young adults surveyed were experiencing at least one GI symptom of a severity greater than normative levels. Both the Mild and Moderate GI groups demonstrated a similar configuration of symptoms with significantly the higher levels of pain, gas/bloating, and nausea/vomiting compared to the Normal group. The configuration of symptoms did not map discretely onto the Rome IV diagnostic categories for Bowel Disorders, such as IBS with predominant Diarrhea or Functional Constipation as might be expected. Rather, the emergent groups suggest that Bowel Disorders occur on a continuum of severity across multiple symptom areas. Mild to moderate GI symptoms appear to emerge at much earlier ages and are more frequent than previously documented. It is recommended that health service providers evaluate individual patterns of “GI health” when young adults present with anxiety and depression, and conversely, they should assess anxiety and depression when they present with GI complaints.

## Background

Functional gastrointestinal disorders (FGIDs) are disorders of gut-brain interaction (DGBI). DGBI are characterized by persistent and recurring gastrointestinal (GI) symptoms that are a result of abnormal functioning of the GI tract and not associated with obvious structural or biochemical abnormalities. DGBI include any combination of the following: motility disturbance, visceral hypersensitivity, altered mucosal and immune function, altered gut microbiota, and altered central nervous system processing [1]. The Rome IV criteria provide a widely accepted diagnostic taxonomy containing 6 primary DGBI domains for adults including: 1.) Esophageal Disorders, 2.) Gastroduodenal Disorders, 3.) Bowel Disorders, 4.) Centrally Mediated Disorders of GI Pain, 5.) Gallbladder and Sphincter of Oddi Disorders, and 6.) Anorectal Disorders. Each DGBI is classified based on the patient’s report of symptom type and severity. One of the most studied domains is Bowel Disorders. This domain is further separated into 6 subcategories including an irritable bowel syndrome (IBS) subcategory, the most frequently diagnosed GI disorder [2]. Some have criticized the Rome IV criteria and prefer to describe DGBI as a “spectrum of chronic GI disorders with combinations of symptoms … existing on a continuum rather than as discrete disorders” [3]. Multiple studies support this dimensional approach, providing scientific evidence that patients can transition from one disorder to another and may receive multiple diagnoses [2–5]. Based on recent scientific knowledge regarding the multifactorial etiology of DGBI and the non-specific and stigmatizing nature of the term “functional,” the Rome IV Foundation has recommended that FGIDs be referred to as DGBI. Nonetheless, since the acronym FGID has been embedded in gastroenterological studies, our literature review will remain consistent with terminology used by previous authors’ empirical work.

A study within a general US adult population (*n* = 71,812, ages 18–65) used the National Institutes of Health (NIH) Patient-Reported Outcomes Measurement Information System GI scales (PROMIS-GI) to evaluate the prevalence of eight overarching GI symptom domains: abdominal pain, bloating/gas, bowel incontinence, constipation, diarrhea, swallowing, reflux, and nausea/vomiting [6]. Sixty-one percent of their sample endorsed at least one symptom within the past 7 days. Of those, 58.4% indicated they experienced two or more symptoms concurrently. A third of their sample population experienced reflux/heartburn, making it the most prevalent symptom. One quarter reported abdominal pain and a fifth of their participants’ experienced bloating, diarrhea, and constipation. This study included emerging adults in their population sample, finding that over 54% (*n* = 6954) reported the occurrence of at least 1 GI symptom within the past week. However, further descriptions of GI symptoms within emerging adults were not provided.

Generally, emerging adults (age 18–25) are viewed as a physically healthy cohort [7] and consequently often overlooked in current GI health research. More recent epidemiological studies suggest that FGIDs are increasing in emerging adults [8–10]. As many as 65% of emerging adults are experiencing symptoms [11] and approximately one third are seeking medical care [12]. Of all the FGID syndromes, the most studied in emerging adults is IBS. According to the American College Health Association (ACHA) National College Health Assessment II national survey for the Fall 2017 semester, 3.2% of the undergraduate students surveyed (*n* = 5789) had been diagnosed by a healthcare professional of having IBS [13]. Another study evaluated the frequency of self-reported IBS symptoms in college students demonstrating that 34% of the sample (*n* = 508, mean age: 22.0+/− 2.8 yrs) experienced clinical levels [12]. This previous research demonstrated a high incidence of IBS in the emerging adult population but is limited in that it does not capture a broader range of general GI distress or other clinical symptomatology.

Emerging adulthood marks the shift from being dependent on a care provider to taking independent responsibility for seeking medical care [14]. Research indicate this population have decreased adherence to medication and attend fewer physician appointments [9, 15]. Furthermore, this period establishes fundamental health and self-care behaviors that carry forward into adulthood [16, 17]. Adverse health behaviors have been observed in the amount of sleep, cigarette use, drinking, exercise, and eating habits of emerging adults [15, 17, 18].

The current understanding of DGBI are supported by a biopsychosocial model [1], which places equal value in researching the patient’s reported experience of illness with the physical indicators of disease [19]. Additionally, researchers have identified a bi-directional communication pathway between the central nervous system and the GI tract, termed the gut-brain axis [1, 20]. The gut-brain axis suggests that changes in either the central nervous system or gut can disrupt the balance of the other. Therefore, psychosocial factors impacting the gut-brain axis could enhance the risk of developing GI symptoms, symptom severity, and affecting treatment outcomes [1, 20]. At present, the psychosocial factors involved include but are not limited to environmental, cultural, and psychosocial factors, including the composition of an individual’s gut microbiome, diet, and nutrition [20].

An environment with chronic and high levels of life stress has proven to be one of the strongest factors for developing FGIDs [19]. Emerging adults are especially susceptible to chronic stress as they transition into adulthood [21]. Stress provoking environments for emerging adults include attending college and adjusting to new social settings [22]. Consequently, the inability to properly cope with chronic stress frequently results in depression and maladaptive eating behaviors in emerging adults [17]. According to the latest Rome IV overview, psychosocial factors associated with the gut-brain axis that interact with the development and severity of FGIDs include mood disorders (depression and suicide ideation), anxiety disorders, somatization, and cognitive-affective processes [20].

Anxiety disorders are closely associated with the onset and duration of FGIDs. Studies have found that general anxiety disorders (GAD) are directly associated with the biological stress response processes, and as a result, can alter pain tolerance and GI motility [20]. In a sample of 604 college students (age = 20.9 ± 1.5 years), 36.9% endorsed IBS symptoms, according to Rome III criteria, with 13.9% presenting with both IBS and GAD [23]. Additionally, it has been argued that anxiety disorders have a greater impact on the risk, comorbidity, and outcome of IBS than depression [24]. The prevalence of depression was found in 30% of medical-seeking patients presenting with FGIDs [25] with 15 to 38% of clinical patients with IBS presenting with suicidal ideation [26], while anxiety disorders were revealed in 30–50% of clinical patients with FGIDs [20]. Only a few studies have evaluated GI symptoms and depression in an emerging adult population. One study with emerging adults found that 13.6% (*n* = 773) of their sample reported moderate to major depression [27]. The comorbidity of depression and anxiety can be associated with poor health outcomes and inferior quality of life [28, 29]. Experiencing chronic GI symptoms can also result in consequences for overall health-related quality of life (HRQoL), i.e. “… one’s general well-being, daily function status, and sense of control over the symptoms” (p. 1273) [1]. Studies have shown that HRQoL was significantly lower in individuals with IBS than healthy individuals [30]. However, studies concerned with health outcomes in emerging adults are very limited.

### Problem statement: defining patterns of GI symptoms in young adults

The purpose of this study was to identify common GI symptom groups within emerging adults based on the National Institutes of Health (NIH) Patient-Reported Outcomes Measurement Information System GI symptom scales (PROMIS-GI) which is freely available at www.healthmeasures.net. A secondary goal was to relate the emergent groups to the Rome IV DGBI symptom domains. A tertiary goal was to identify psychosocial comorbidities within these groups. The use of the PROMIS-GI scales afforded this study with a means to measure a broad range of GI functioning and symptom levels within a general emerging adult population group. To date, there is no comprehensive study exploring general GI functioning in the emerging adult population using the PROMIS-GI symptom scales. To identify common GI symptom patterns, a latent class analysis approach was employed. Latent class analysis (LCA) is a statistical method that allows the researcher to use a set of observed variables to identify hidden but meaningful patterns resulting in homogenous groups of participants (latent classes) [31]. Ideally these groups would represent symptom profiles corresponding to different DGBI diagnostic categories.

## Method

### Participants

Undergraduates enrolled in introductory psychology courses at the University of Central Florida were recruited to participate in ongoing research monitoring physical and psychological health for course credit. Introductory psychology is a required course in the general education curriculum for most majors at this university. Therefore, all college students and majors were well represented. Eligibility criteria excluded vulnerable populations, required participants to be between the age of 18 and 25 years, and able to complete an online questionnaire in the English language. This study was approved by the UCF Institutional Review Board (IRB).

### Measures

The online survey totaled 198 questions and the survey took approximately 30 min to complete. The survey was delivered using Qualtrics software (Qualtrics, Provo, UT).

#### Demographic assessment

Demographic information collected in this study included age, sex, race/ethnicity, marital status socioeconomic status, housing, BMI, and drug use. See Table 1 for a description of study participants on these variables.

**Table 1:** Descriptive Characteristics of Participants

Variable	n	%
**Age**		
18	515	53.9%
19	222	23.2%
20	94	9.8%
21	52	5.4%
22	33	3.5%
23	19	2.0%
24	10	1.0%
25	11	1.2%
**Sex**		
Male	399	41.7%
Female	557	58.3%
**Race/ethnicity**		
Non-Hispanic white	548	57.3%
Non-Hispanic black	114	11.9%
Puerto Rican	51	5.3%
Mexican-American	14	1.5%
Other Hispanic	108	11.3%
Asians	92	9.6%
American Indian	10	1.0%
Other	19	2.0%
Identified with 2+ ethnicities	81	8.5%
**Living Arrangements**		
On campus	474	49.6%
Off campus	482	50.4%
**Total Household Income**		
0–50,000	362	37.9%
50,001-100,000	295	30.9%
100,001-150,000	166	17.4%
≥ 150,001	133	13.9%
**Health**		
Allergies	300	31.4%
Currently taking antibiotics	48	5.0%
Taking antibiotics past 2 months	197	20.6%
Taking probiotics	89	9.3%
Taking multivitamins	356	37.2%
Currently a smoker	130	13.6%
**Body Mass Index (BMI)**		
Underweight ≤18.5	47	7.9%
Normal weight = 18.5–24.9	629	65.8%
Overweight = 25–29.9	168	17.6%
Obesity = BMI of 30 or greater	112	11.7%

#### The NIH PROMIS-GI symptom scales

**Fig. 1:**
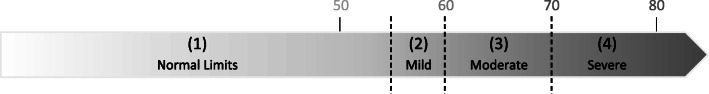
Coding Symptom Severity Range from PROMIS T-Score. *Note*. Symptom severity ratings were based on the recommended PROMIS T-Score ranges, using the mean of 50 and standard deviation (SD) of 10. Normal limits (1) = t-scores < 55; Mild (2) = t-scores between 55 and 60; Moderate (3) = t-scores between 60 and 70; Severe (4) = t-scores > 70. Adapted from http://www.healthmeasures.net/score-and-interpret/interpret-scores/promis

The PROMIS-GI has been validated in studies as an effective PRO measure to be used in both clinical and general populations [32]. The PROMIS-GI scales evaluate eight GI symptom domains, of which this study focused on six: abdominal pain (6 items), gas/bloating (12 items), diarrhea (5 items), constipation (9 items), gastroesophageal reflux (GER) (13 items), and nausea/vomiting (4 items). Individuals’ scores are provided as a T-score metric with 50 representing the U.S. general population mean with a standard deviation (SD) of 10 [32]. This means the higher the T-score, the greater the severity of the symptom. T-scores were calculated by the scoring service available via the PROMIS website. T-scores were then converted into GI symptom severity levels using the suggested ranges of mild (T-scores between 55 and 60), moderate (T-scores between 60 and 70), and severe (T-scores above 80) as demonstrated in Fig. 1.

#### Patient health questionnaire (PHQ-9)

To evaluate self-reported symptoms of depression, the Patient Health Questionnaire (PHQ-9) was administered. This instrument consists of 9 items, scored 0 (not at all) to 3 (nearly every day), with a total summary score of 27. Validated cut-off points include scores above 10 considered mild symptoms, and scores of 15 or greater indicating moderate to severe symptoms [33]. The PHQ-9 has been validated with other widely used instruments [33].

#### Generalized anxiety disorder 7-item scale (GAD-7)

To evaluate self-reported symptoms of anxiety, the Generalized Anxiety Disorder Screener (GAD-7) was administered. This quantifies levels of general anxiety experienced within the past 2 weeks using a set of 7 questions [34]. The GAD-7 scored each question from 0 (not at all) to 3 (nearly every day) with a total score of 21. Summary scores of 5, 10, and 15 are frequently used as threshold values for mild, moderate and severe anxiety [34]. The GAD-7 was constructed using existing GAD criteria from the Diagnostic and Statistical Manual of Mental Disorders, Fourth Edition (DSM-IV [34]. It has been validated in multiple studies [35].

#### 36-item short form health survey 1.0 scales (SF-36)

The SF-36 was administered to measure the HRQoL status of participants. The SF-36 contains eight scales: Physical Functioning, Role Limitations Due to Physical Health, Role Limitations Due to Emotional Problems, Energy/Fatigue, Emotional Well-Being, Social Functioning, Bodily Pain, and General Health Perceptions [36].

#### Validity check items (VCheck)

Nine items were dispersed throughout the survey to identify random, careless, or inattentive responding. Example items include, “For this item, please select Yes,” and “For this item, please select Never.” Participants were excluded from the sample if they answered one or more of these questions incorrectly. We have used this approach successfully in our laboratory with several studies and the use of VCheck items has a long tradition in psychological testing beginning with the Minnesota Multiphasic Personality Inventory and the Millon Clinical Multiaxial Inventory [37].

### Statistical analysis

Severity ratings from the PROMIS-GI symptoms scales were analyzed in LatGold v5.1 (Statistical Innovations Inc.), a latent class analysis software package. LCA methods have the same goal as traditional cluster analysis, in that both attempt to create the largest between-cluster and smallest within-cluster differences. However, unlike standard cluster methods, LCA uses a probabilistic model-based approach rather than distance measures of dissimilarity [38]. The ideal model was based on appropriate model fit, the number of individuals per class, the certainty of being assigned to one class (membership probability), and significant difference between classes [39]. Class differences based on psychosocial factors were then explored using multivariate analysis of variance (MANOVA) analysis. Groups that differed significantly were compared at a pair level using the Tukey’s Honestly Significant Difference (Tukey’s HSD) test. A *p* value of < 0.05 was considered statistically significant. Both MANOVA and Tukey’s HSD tests were conducted in SPSS (IBM Corp. Released 2017. IBM SPSS Statistics for Windows, Version 24.0. Armonk, NY: IBM Corp.).

## Results

The online survey was made available to 1786 introductory psychology students over the course of the summer and spring 2018 semesters (May to December 2018). There were 1238 total respondents to the survey announcement and 213 of these were eliminated because they exceeded the VCheck item criteria for random responding, and 69 respondents were eliminated because of missing data for entire scales. Single missing items were replaced by the series mean. The final study sample totaled 956 emerging adults between the age range of 18 and 25 (*M* = 19.0, *SD* = 1.5) with 58.3% identifying as female, and 57.3% identified as Caucasian. To evaluate the presence of GI symptoms within the emerging adult sample group, the T-scores derived from the PROMIS-GI symptoms scales were assigned a rating of 1 through 4, as illustrated in Fig. 1, marking symptom severity. Symptom prevalence was assessed using the severity scores. Using these ratings, frequencies were calculated using SPSS. As presented in Table 2, 36.4% of the emerging adult sample group presented with at least one GI symptom within the past 7 days. Next, latent class analysis (LCA) was conducted using the assigned symptom severity scores of 1–4 for each PROMIS-GI scale.

**Table 2:** Frequency of GI Symptom Severity Found across all Participants (*n* = 956)

GI Symptom Severity (Rating)	Belly Pain	Constipation	Diarrhea	Gas/Bloating	Nausea/Vomiting	Reflux/Heartburn
**Within Normal Limits (1)**	750 (78.5%)	845 (88.4%)	870 (91%)	608 (63.6%)	699 (73.1%)	891 (93.2%)
**Mild (2)**	99 (10.4%)	77 (8.1%)	53 (5.5%)	247 (25.8%)	142 (14.9%)	49 (5.1%)
**Moderate (3)**	91 (9.5%)	34 (3.6%)	32 (3.3%)	99 (10.4%)	105 (11%)	15 (1.6%)
**Severe (4)**	16 (1.7%)	–	1 (0.1%)	2 (0.2%)	10 (1%)	1 (0.1%)
**Presenting with Symptoms**	**206 (21.6%)**	**111 (11.7%)**	**86 (8.9%)**	**348 (36.4%)**	**257 (26.9%)**	**65 (6.8%)**

*Note.* Levels of severity were interpreted using the threshold range guidelines developed by the NIH to be used with their PROMIS measures. Within Normal Limits = T-scores < 55; Mild = T-Scores between 55 and 60; Moderate = T-Scores between 60 and 70; Severe = T-scores > 70. There was no endorsement for severe constipation within the sample

A baseline model was created using a 1-Class (latent) cluster model [40]. Classes were subsequently added and compared to the baseline 1-Class model. Model sizes with up to 7 classes were calculated as there were no previous studies that suggested the number of classes for conducting a latent class analysis using the PROMIS-GI scales.

Table 3 provides an overview of the various information criteria considered in determining the best model fit. The information criteria consisted of the likelihood ratio chi-squared statistic (L2) and Bayesian Information Criterion (BIC) with lower values indicating improved model prediction of the data (p.69) [41]. The L2 statistic calculates the similarity between model-based estimated frequencies and observed frequencies with smaller values indicating better model fit. The BIC accounts for model complexity and endorses model parsimony of the latent classes, and when using sample sizes larger than 500, proves to be a superior indicator to model fit compared to all other information criteria (p. 563) [42]. A more formal assessment of the model holding true for the population is determined by the *p*-value with *p* <  0.05 indicating a poor model fit. Due to some of the GI symptom severity levels containing small group sizes, a bootstrapping method was used to better assess the global fit of the model [42]. Additionally, entropy R-squared was evaluated for quality of membership classification with values closest to 1 indicating improved probability of an individual belonging to just one class [31]. Individual class sizes below 3% were considered too small for this study. Accordingly, the 4-Class model (and higher) were thus eliminated.

**Table 3:** Summary of Statistical Model Fit Statistics Used for Model Selection

Model	BIC	L^**2**^	***df***	***p*** ^a^	Entropy	Class Error
Baseline 1-Class Model	6798.2738	1185.6210	940	1.00	1.00	0.0000
2-Class Model	**6339.2847**	603.0833	922	0.0020	**0.7033**	**0.0621**
3-Class Model	6344.9257	**485.1760**	904	**0.0980**	0.6426	0.1101
4-Class Model	6426.8327	443.5345	886	0.0640	0.6548	0.1139
5-Class Model	6518.9844	412.1377	868	0.0119	0.6022	0.1637
6-Class Model	6624.2116	393.8164	850	0.0142	0.6939	0.1347
7-Class Model	6722.1687	368.2251	832	0.0103	0.7045	0.1332

*Note.* Comparison between the 2-Class and 3-Class are shown with values in bold indicating optimal values. The 4-Class and higher models did not meet the minimum group size criteria. *BI* Bayesian information criterion; *L*^*2*^ Likelihood-ratio; *df* Degrees of freedom; *p* p-value; Entropy = quality of predicting model classification with values closer to 1 preferred

^a^ *p* value calculated using bootstrap method

The 2-Class model had the lowest BIC; however, conducting a conditional bootstrap analysis revealed that the 3-Class model showed a statistically significant improvement over the 2-Class model (*p* <  0.05) for overall model fit, thus the 3-Class model was selected.

### Describing the latent classes (groups)

**Fig. 2:**
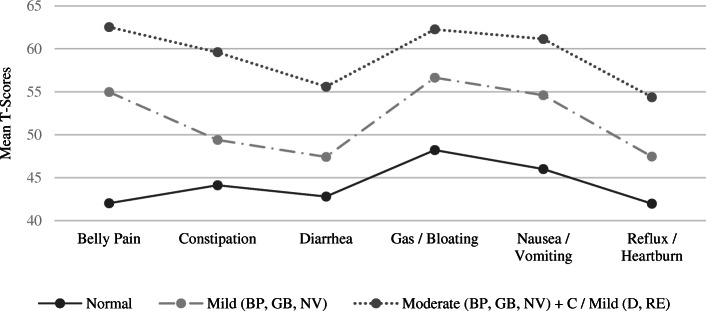
3-Class Model Profile Plot Using Conditional Mean T-Scores per Class. *Note*. PROMIS T-Score ranges for GI symptoms include Normal limits = < 55; Mild = between 55 and 60; Moderate = between 60 and 70; Severe = > 70

Differences between classes are graphically illustrated in Fig. 2 based on the T-Score means for each class. The differences between these classes were statistically significant (*p* < .001) for each symptom domain. It was concluded that the 3-Class model adequately identified three unique latent classes that were informative to the study and could be defined based on their GI symptom patterns. The three classes or groups were described as Normal (649 individuals, 67.89%), Mild (257 individuals, 26.88%), and Moderate (50 individuals, 5.2%). Visual inspection of the profile of scale scores in Fig. 2 indicates that symptom severity marked the main difference between these groups. The Mild group fell into what is likely a pre-clinical range with 3 symptom scores .5 *SD* above the normative population mean, while the moderate group was probably in the clinical range with 4 symptom scores 1 *SD* above the population mean. Both the Mild and Moderate GI groups demonstrated the highest relative symptom elevations in pain, gas/bloating, and nausea/vomiting. Both groups evidenced higher levels of nausea/vomiting than would be expected with typical IBS diagnoses. Taken together, the symptom patterns for the Mild and Moderate groups were consistent with IBS mixed or unclassified subtypes following the Rome IV diagnostic criteria.

### Group differences on psychosocial and HRQoL factors

Based on previous literature, this study hypothesized that the groups would differ on psychosocial factors with a decrease in psychosocial functioning as levels of GI symptoms increase (Mild and Moderate classes). The evaluation of psychosocial factors and HRQoL was conducted subsequent to independently establishing the GI symptoms groups so as to not confound the two research questions. There was a statistically significant main effect for group on the combined dependent variables, *F* (8, 1890) = 13.237, *p* < .001, Pillai’s V = .246, partial η^2^ = .123. Follow-up univariate ANOVAs were run, showing a statistically significant main effects for group across all Psychosocial and HRQoL measures. Post hoc Tukey pairwise comparisons were then conducted to evaluate the differences in mean Psychosocial and HRQoL scores between group. The univariate and post hoc comparisons are presented in Table 4. To summarize, the Mild group demonstrated significantly higher scores on the PHQ-9 and GAD-7 than the normal group. The Mild group also showed significantly lower scores on all SF-36 scales when compared to the normal group. In turn, the Moderate group demonstrated significantly higher scores on the PHQ-9, GAD-7, and Bodily Pain compared to the Mild group. The Moderate group also showed significantly lower scores on the SF-36 Role Limitations Due to Physical Health and General Health Perceptions compared to the Mild group.

**Table 4:** Comparison of GI Symptom Groups and Psychosocial and HQoL Variables

	Latent classification GI group Mean (SD)			ANOVA main effect			Post hoc tests Mean difference significance		
Variables	Normal (***n*** = 648)	Mild (***n*** = 258)	Moderate (***n*** = 50)	F	p	η^**2**^	Normal (v) Mild	Normal (v) Moderate	Mild (v) Moderate
PHQ-9	4.90 (4.57)	8.47 (5.63)	10.30 (6.525)	47.924	< 0.001	.092	***	***	*
GAD-7	3.95 (4.43)	7.57 (5.58)	10.60 (6.45)	54.438	< 0.001	.103	***	***	***
SF-36 SCALES									
PHYSICAL FUNCTION	94.23 (14.49)	91.76 (15.63)	88.70 (16.19)	5.013	< 0.01	.010		*	
ROLE FUNCTION/ PHYSICAL	91.63 (20.00)	84.50 (28.02)	70.00 (38.80)	23.442	< 0.001	.047	***	***	***
ROLE FUNCTION/ EMOTIONAL	71.25 (36.75)	53.62 (38.08)	46.67 (34.34)	27.561	< 0.001	.055	***	***	
ENERGY/ FATIGUE	55.02 (18.44)	44.69 (16.59)	40.40 (20.15)	40.147	< 0.001	.078	***	***	
EMOTIONAL WELL-BEING	66.34 (17.54)	56.20 (18.60)	49.92 (22.16)	42..205	< 0.001	.081	***	***	
SOCIAL FUNCTION	83.10 (20.64)	70.00 (25.07	62.75 (24.16)	45.759	< 0.001	.088	***	***	
PAIN	86.82 (15.18)	76.85 (18.72)	64.45 (21.25)	66.402	< 0.001	.122	***	***	***
GENERAL HEALTH	68.28 (16.17)	59.38 (16.20)	52.50 (19.07)	42.971	< 0.001	.083	***	***	*

*Note.* Post-hoc comparisons were evaluated using Tukey’s HSD and are marked according to the degree of significant difference. *PHQ-9* Patient Health Questionnaire; *GAD-7* Generalized Anxiety Disorder; *SF-36* 36-Item Short Form Health Survey 1.0

⁎ *p* < .05. ⁎⁎ *p* < .01. ⁎⁎⁎ *p* < .001

## Discussion

Recent studies have demonstrated that GI symptoms are common in the general population. However, there is limited information on patterns of GI symptoms in emerging adults, individuals between the ages of 18 and 25. Self-reported GI symptoms were assessed using the freely available PROMIS-GI scales. Latent class analyses revealed that 32% of the emerging adults surveyed here experienced one or more GI symptom above normative ranges, and 5.5% of the sample reached levels of GI symptom severity associated with clinical diagnoses. Three latent GI classes or groups were identified, Normal (*n* = 649, 67.89%), Mild (*n* = 257, 26.88%), and Moderate (*n* = 49, 5.2%).

GI symptom severity marked the main difference between the three groups. Specifically, both the Mild and Moderate GI groups demonstrated a similar configuration of symptoms with elevations in pain, gas/bloating, and nausea/vomiting relative to the other symptom domains. The symptom overlap across the Mild and Moderate groups supports the proposition that GI disorders exist on a continuum and that emerging adults can transition from one domain to another in their experience of symptoms (p. 4) [3]. Visual inspection of Fig. 2 while considering the Rome IV diagnostic criteria suggests that the configuration of symptoms for the Mild and Moderate groups were at best consistent with IBS mixed or unclassified subtypes following the Rome IV diagnostic criteria. This conclusion is offered since the chief or peak complaint of participants in the Mild and Moderate groups was not diarrhea or constipation, and the Moderate group reported elevated symptoms in both diarrhea and constipation.

The two previous studies on GI symptoms in emerging adults found even higher rates of GI symptoms in college students. In one, 51.2% of Canadian-based university students endorsed at least one GI symptom [43] and in another study, 65% of Korean-based nursing students reported more than one GI symptom [11]. The high incidence of GI symptoms in this age range is surprising. However, both studies used high achieving college or professional students under high stress [21]. Collectively these findings may reflect a relationship between GI functioning and stress among the other factors discussed.

Previous cluster analytic/LCA studies have combined GI symptom data and other non-GI data such as stress, fatigue, sleep, depression, etc. in their statistical approach to establish diagnostic classes or groups [44, 45]. This has led to groups that are difficult to classify based on Rome IV or other diagnostic criteria because physical and psychosocial symptoms confounded, and causal modeling becomes circular. In the current study, the GI symptom groups were formed first and then individuals were compared on widely used clinical screening measures for depression, anxiety, and HRQoL. The Moderate GI symptom group met the PHQ-9 threshold score of 10 or higher for moderate or severe depression, and the Moderate GI symptom group met the GAD-7 threshold for moderate anxiety levels. This is consistent with other studies that have found that GI symptoms are frequently associated with anxiety and depression [20, 24–27, 30]. For example, previous studies showed 13.9% of their sample presented with both IBS and anxiety [23] and another found 30% of their patients presented with FGIDs and depression [25].

The GI symptoms and associated psychosocial measures found in this study are consistent with the existence of a gut-brain axis communication pathway. The bi-directional communication between the gut and brain is integral in maintaining homeostasis and an imbalance in either can have adverse consequences [46]. Following this theory, psychosocial functioning can excite or suppress the GI system, or GI functioning can excite or suppress psychosocial functioning [20]. This study observed that the level of GI symptom severity was strongly associated with greater impairment in mood, anxiety and HRQoL.

### Limitations

The emerging adult population used here was from a university sample, thus generalizing results to the entire population of emerging adults remains to be determined. However, while the sample was from a general psychology class, it fulfills the social science requirement of all students, regardless of their major. Thus, while participants were more educated than the general population in this age range, they were broadly represented within the age range. The participants were not presenting for medical treatment and did not receive a medical exam or diagnosis from a physician. Therefore, we cannot estimate the number of participants who have “organic” conditions such as infections, ulcers, etc. This study was a cross-sectional study and thus causation could not be determined. The PROMIS-GI Belly Pain scale does not specifically ask if the pain occurs during bowel movements, however this scale is imbedded with other scales asking about bowel functioning. Two PROMIS-GI scales were excluded from the survey measures; one focused on disrupted swallowing and the other on bowel incontinence. Furthermore, several studies suggest that GI symptom severity increases during menstruation [47], however, this came to our attention after the study began and we did not account for possible interactions between menses and belly pain.

### Future directions

Including all PROMIS-GI measures in future research would provide a broader scope of GI functioning. Furthermore, additional insight will be gained by comparing the GI symptom groups on other demographic and psychosocial measures. Of particular interest would be comparisons of GI functioning across racial/ethnic groups such as Hispanic/Latino and Black participants because there was not enough statistical power to make such contrasts here. Future studies should also consider measuring GI and psychosocial variables over repeated intervals with a time-series design. That way possible cause and effect relationships may be determined. Future research evaluating GI symptoms in young adults should include more details about the duration of symptoms and past healthcare seeking measures to determine the likelihood that this population has sought treatment for their GI or psychosocial symptoms. If a larger sample were available, future research could perform an LCA or other statistical clustering approach using just respondents with mild, moderate, or severely elevated symptoms. This may produce groupings with configurations of symptoms more closely aligned with the Rome IV categories. Additionally, stool diaries, and assessment about menses related pain should be considered in future research.

## Conclusions

This study demonstrated that approximately a third of young adults surveyed were experiencing at least one GI symptom above normative levels. Both the Mild and Moderate GI groups demonstrated a similar configuration of symptoms with the highest relative elevations in pain, gas/bloating, and nausea/vomiting. The configuration of symptoms did not map discretely onto the Rome IV diagnostic categories for Bowel Disorders, such as IBS with predominant Diarrhea or Functional Constipation as might be expected. Rather, the emergent groups suggest that Bowel Disorders occur on a continuum of severity across fluctuating symptom areas. Hypothetically, if a categorical approach were applied, the Mild and Moderate groups would likely be classified as IBS mixed or unclassified subtypes since participants reported both diarrhea and constipation over the previous 2 weeks. Mild to moderate GI symptoms appear to emerge at much earlier ages and are more frequent than previously documented. Based on this study’s findings, it is recommended that health service providers evaluate individual patterns of “GI health” when young adults present with anxiety and depression, and conversely, they should assess anxiety and depression when they present with GI complaints.

## Data Availability

The dataset used in the current study is available at the ResearchGate repository for Jeffrey E. Cassisi in the file: GI Demographic and Cluster Analysis Data.Sav. DOI: 10.13140/RG.2.2.16826.75209 https://www.researchgate.net/publication/338951233_Demographic_and_Cluster_Analysis_Data

## Abbreviations

ACHA: American college health association
ANOVA: Analysis of variance
BIC: Bayesian information criterion
BMI: Body mass index
df: Degrees of freedom
DGBI: Disorders of gut-brain interaction
FGID: Functional gastrointestinal disorder
GAD-7: Generalized anxiety disorder 7-item (GAD-7) scale
GI: Gastrointestinal
HRQoL: Health related quality of life
IBS: Irritable bowel syndrome
LCA: Latent class analysis
L^2^: Likelihood-ratio
MANOVA: Multivariate analysis of variance
NIH: National institutes of health
p: *p*-value
PHQ-9: Patient health questionnaire 9
PROMIS-GI: Patient-reported outcomes measurement information system-GI symptom scales
SF-36: 36-Item short form health survey v1
Tukey’s HSD: Tukey’s honestly significant difference test
UCF: University of Central Florida
